# Delirium prevalence, diagnostic uncertainty and outcomes in ORCHARD-EPR: validation against prospective reference cohorts

**DOI:** 10.1093/ageing/afaf284

**Published:** 2025-10-16

**Authors:** Emily Louise Boucher, Jasmine Ming Gan, Nicola Georgia Lovett, Sarah Catherine Smith, Sasha Shepperd, Sarah Tamsin Pendlebury

**Affiliations:** University of Oxford, Wolfson Centre for Prevention of Stroke and Dementia, Nuffield Department of Clinical Neurosciences, Wolfson Building, Headington, Oxford, United Kingdom; University of Oxford, Wolfson Centre for Prevention of Stroke and Dementia, Nuffield Department of Clinical Neurosciences, Wolfson Building, Headington, Oxford, United Kingdom; Oxford University Hospitals NHS Foundation Trust, Departments of General (Internal) Medicine and Geratology, Oxford, Oxfordshire, United Kingdom; Oxford University Hospitals NHS Foundation Trust, Departments of General (Internal) Medicine and Geratology, Oxford, Oxfordshire, United Kingdom; University of Oxford, Nuffield Department of Population Health, Richard Doll Building Old Road Campus, Oxford OX3 7LF, United Kingdom; University of Oxford, Wolfson Centre for Prevention of Stroke and Dementia, Nuffield Department of Clinical Neurosciences, Wolfson Building, Headington, Oxford, United Kingdom; Oxford University Hospitals NHS Foundation Trust, Departments of General (Internal) Medicine and Geratology, Oxford, Oxfordshire, United Kingdom; NIHR Oxford Biomedical Research Centre, John Radcliffe Hospital, Headley Way, Headington, Oxford OX3 9DU, United Kingdom

**Keywords:** delirium, electronic health records, diagnostic uncertainty, dementia, reference standard, older people

## Abstract

**Background:**

Delirium is underdiagnosed in older inpatients. We determined clinically ascertained delirium prevalence, characteristics and outcomes after implementation of mandatory cognitive/delirium screening via the electronic patient record (EPR) compared to previously acquired prospective cohorts.

**Methods:**

Acute general medicine patients aged ≥70 years in two separate cohorts were included (i) the Oxford Cognitive Comorbidity, Frailty and Ageing Research Database (ORCHARD-EPR, 2017–2019) and (ii) prospective cohorts (2010–2018). The ORCHARD-EPR cognitive screen included the Confusion Assessment Method, and 10-point Abbreviated Mental Test (AMT) with delirium diagnosis = certain/uncertain/no. In the prospective cohorts, delirium was diagnosed by Diagnostic and Statistical Manual of Mental Disorders, 4th Edition criteria. Odds ratios for in-hospital mortality were adjusted for age, sex, comorbidity and illness severity.

**Results:**

Among 18 614 ORCHARD-EPR patients (mean/SD age = 82.9/7.4 years, dementia = 20%), certain delirium (delirium = yes or ICD-10 delirium code) was present in 3077/18 614 (17%,95%CI 16%–17%) of whom 1199/3077 (39%) had dementia, and uncertain delirium (delirium = uncertain and no ICD-10 code) in 2007 (11%,10%–11%) of whom 773/2007 (39%) had dementia. In the prospective cohorts (*n* = 731, mean/SD age = 82.7/7.1 years, dementia = 21%), 277 (38%, 34%–42%, both *P* < 0.001) had delirium of whom 97 (35%) had dementia. Frailty, AMT < 8, infection and hyponatraemia (Standardised Mean Difference-SMD all >0.1) but not comorbid dementia (SMD = 0.009) were more common in certain vs uncertain delirium. Excess mortality was similar in certain and uncertain delirium and the prospective cohorts.

**Discussion:**

Delirium diagnostic uncertainty was frequent particularly in the absence of key delirium risk factors and delirium prevalence, even combining both certain and uncertain cases, was lower than in the prospective cohorts. Nevertheless, co-occurrence with dementia and outcomes were similar indicating validity of screening and research potential of ORCHARD-EPR.

## Graphical Abstract

**Graphical Abstract:**
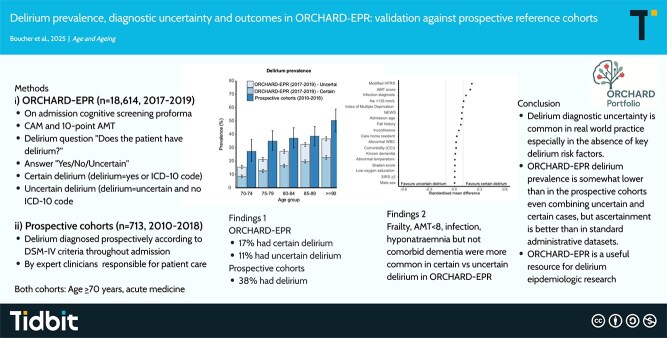

## Key Points

- Following electronic patient record (EPR)-implementation of cognitive screening, delirium ascertainment, cognitive test scores and outcomes were broadly comparable to reference standard prospective cohorts.
- Delirium diagnostic uncertainty was common in real-world clinical practice especially in the absence of key delirium risk factors.
- Clinicians were more certain about assigning a delirium diagnosis in those with infection, low cognitive test score, frailty or hyponatraemia.
- Comorbid dementia did not increase delirium diagnostic uncertainty.
- Oxford Cognitive Comorbidity, Frailty and Ageing Research Database-EPR will facilitate epidemiologic studies of delirium combining the large inclusive scale of administrative health data with the sensitivity of small prospective studies.

## Introduction

Delirium is an acute neuropsychiatric syndrome characterised by disturbance in attention, cognition and awareness that is often precipitated by acute illness or trauma. [1] Delirium affects up to a third of older hospital patients and is associated with mortality, complications, prolonged hospitalisation and excess costs [2–4]. Current guidelines recommend screening for delirium and other cognitive frailty to improve patient care but recognition and documentation is poor outside research settings [5, 6]. Electronic patient records (EPRs) bring the opportunity to implement screening at scale but there are few real world studies comparing delirium detection following implementation with a local reference standard (Appendix 1 and Appendix Table 1) [1, 6]. Screening by clinical staff may not be comparable to assessment by researchers, particularly where collateral information or medical records are unavailable.

In addition to improving patient care in real time, successful implementation of routine delirium screening would provide high quality real world, digital data for quality improvement, service planning, policy and research [1, 5]. At present, standard hospital administrative datasets are limited by very low delirium ascertainment [7, 8]. EPRs could provide cognitive screening data at scale without the selection bias inherent in studies requiring informed patient consent [9].

We previously validated and implemented a pragmatic cognitive screen to capture all forms of cognitive frailty including cognitive deficits and their severity, delirium and pre-existing dementia as part of the routine clinical assessment of older hospital patients [2, 10, 11]. The screen included the Confusion Assessment Method (CAM) [12] and 10-point Abbreviated Mental Test (AMT) [13] and required clinicians to document delirium and record diagnostic uncertainty. The screen was initially introduced via a paper clerking proforma (2013–15) [2] and then implemented via a structured EPR-embedded proforma (2015) supported by a multicomponent intervention including education and compliance feedback using electronic data capture [8, 10].

In the current study, we compared clinically ascertained delirium in large scale structured EPR data following implementation of the cognitive screen with the reference standard of prospectively acquired cohorts from the same setting in which delirium was diagnosed by expert clinicians using the DSM-IV criteria. We also explored the characteristics and outcomes of patients with certain versus uncertain delirium diagnosis in the EPR data including after stratification for comorbid dementia status.

## Methods

### Setting

Oxford University Hospitals NHS Foundation Trust (OUHFT) consists of four general hospitals providing all acute secondary care in Oxfordshire, UK with a catchment area of >800 000 people. The Oxfordshire population is older than the England average, with an urban/rural mix in line with England as a whole. The current study used two separate, mutually exclusive cohorts from OUHFT acute general (internal) medicine acquired with different methodology during overlapping time periods as described below. The study was reported according to STROBE guidelines [14].

### Study design

#### Oxford cognitive comorbidity, frailty and ageing research database-electronic patient records

The first cohort comprised routinely acquired individual pseudonymised EPR data including cognitive screening (see below) contained within the Oxford Cognitive Comorbidity, Frailty and Ageing Research Database (ORCHARD-EPR) [15]. For the current study, we included all unplanned admissions in patients aged ≥70 years to general (internal) medicine with length of stay $\boldsymbol{\ge}$1 day between 1 January 2017 and 31 December 2019 (from full EPR implementation up to the Covid-19 pandemic, Appendix Figure 1).

#### Prospective cohorts used as the reference standard

We compared ORCHARD-EPR data with our previously acquired prospective cohorts in which delirium (occurring at any time during admission) and other cognitive frailty (dementia, low AMT) had been carefully ascertained [2, 3, 8, 16]. Over six eight-week cycles (2010–2018) consecutive patients aged ≥70 years had the cognitive screen administered by the admitting resident doctor. Patients were seen at least every other day until discharge, death, or transfer by STP/SCS who diagnosed delirium according to Diagnostic and Statistical Manual of Mental Disorders, 4th Edition (DSM-IV) criteria based on all available information. Pre-existing dementia diagnosis was ascertained from the patient, relatives, and medical records.

Only first admissions were included in ORCHARD-EPR and the prospective cohorts (ie readmissions were excluded). Admission episodes included in the 2017–2018 prospective cohorts were excluded from the ORCHARD-EPR dataset. ORCHARD-EPR was approved by the regional ethics committee with a waiver of individual consent although individuals could opt-out (REC reference: 17/SC/0184; which also covered use of the prospective cohort data).

### Cognitive screen

The cognitive screen has been described previously (Appendix 2) [2, 8, 10]. Briefly, the screen included the 10-point AMT, or reason for untestability selected from a menu (eg too unwell, uncooperative) and the question: ‘Does the patient have delirium?’ with a choice of answer (yes/no/uncertain). The CAM items were displayed alongside the delirium question to inform diagnosis but the individual CAM items were not scored. Training for resident doctors was provided by STP with delirium diagnosis made as part of a holistic clinical assessment and not solely on the basis of the CAM. Proforma completion was mandated in untestable as well as testable patients and partial completion was not possible.

The inclusion of ‘uncertain’ as a response to the delirium question reflected real-world clinical practice where there may be diagnostic uncertainty, especially at initial assessment. The electronic screen was triggered automatically on admission for all patients aged ≥70 years for completion by the clerking resident doctor [10] but could also be completed ad hoc during admission.

### Delirium definitions in ORCHARD-EPR

Delirium in ORCHARD-EPR was defined according to the assessor’s answer to the delirium question together with administrative ICD-10 delirium coding as follows:

i) Certain delirium (delirium = ‘yes’ or ICD-10 delirium code)
ii) Uncertain delirium (delirium = ‘uncertain’ and no ICD-10 delirium code)

Delirium ICD-10 coding has good sensitivity and high specificity for delirium in our institution [8]. We included ICD-10 coding to capture delirium episodes where the screen was not performed or the answer to the delirium question was no/uncertain but a delirium diagnosis was recorded in free text and subsequently coded (Appendix Figure 2, Appendix 3) [15]. Dementia was defined as dementia = ‘yes’ on the cognitive screen or ICD-10 dementia code.

### Covariates

Covariates were obtained from ORCHARD-EPR or from paper records. Illness severity/acuity was measured using the National Early Warning Score (NEWS) and the systemic inflammatory response syndrome (SIRS) [17, 18]. ICD-10 codes were used to calculate a modified Hospital Frailty Risk Score (HFRS) excluding coded delirium [19] and the Charlson co-morbidity index (CCI) [20].

**Table 1:** Comparison of clinical characteristics of ORCHARD-EPR (2017–2019) and prospective cohorts (2010–2018)

Characteristic	ORCHARD-EPR 2017–2019, N = 18 614	Prospective reference cohorts 2010–2018, N = 731	*P*-value^a^
Age	82.9/7.4	82.7/7.1	0.6
Female sex	9774 (53%)	389 (53%)	0.7
CCI	10.5 (10.4)	10.7 (10.7)	0.9
***Frailty markers***			
HFRS	7.9 (6.3)	6.9 (6.0)	<0.001
Fall history	6306/15 065 (42%)	333/730 (46%)	0.045
Incontinence	3471/15 097 (23%)	231/729 (32%)	<0.001
Braden score^b^	17.9/3.6	17.9/3.6	>0.9
***Observations and labs***			
SIRS ≥2	7146/18,331 (39%)	189/496 (38%)	0.7
Low oxygen saturation	5374/18,585 (29%)	147/513 (29%)	0.9
CRP >6 mm/L	13,077/16,994 (77%)	396/538 (74%)	0.07
Na <135 mm/L	5768/18,394 (31%)	184/581 (32%)	0.9
LoS (days)	3 (1, 9)	4 (2, 10)	0.066
In-hospital death	1815/18,614 (9.8%)	74/731 (10%)	0.7

Numbers are mean/SD or n (%), except for length of stay for which Median (IQR) is shown.

HFRS = Hospital Frailty Risk Score. Incontinence = Incontinence or urinary urgency. SIRS = Systemic Inflammatory Response Syndrome criteria. Low oxygen saturation = <95% (on room air), CRP = C-Reactive Protein, Na = Serum Sodium, LoS = Length of stay in hospital

^a^T-test; Pearson’s Chi-squared test, Fisher’s exact test or Wilcoxon rank sum test (for length of stay only).

^b^ORCHARD-EPR N = 16,751, Reference Cohorts = 483. CCI = Charlson Comorbidity Index.

### Outcomes

In-hospital death was determined for ORCHARD-EPR and the prospective cohorts. Length of stay >7 days, delayed discharge, discharge other than to home, readmissions within 30 days for people who survived to discharge and mortality to one year (Office of National Statistics) were determined for ORCHARD-EPR stratified by comorbid dementia status.

### Analysis

Descriptive statistics for demographics, patient characteristics and outcomes were calculated. Prevalence and 95% confidence intervals of certain and uncertain delirium in ORCHARD-EPR and of DSM-IV diagnosed delirium in the prospective cohorts, and of dementia, were calculated using Wilson’s method for binomial proportions. ORCHARD-EPR delirium and dementia prevalence was calculated using the entire cohort as the denominator (ie including both patients with and without a completed cognitive screen or ICD-10 coding for delirium/dementia). Patients without a completed cognitive screen or ICD-10 code were assigned as no cognitive frailty (ie no delirium, no dementia, normal AMT). In a subsequent sensitivity analysis, delirium and dementia prevalence were calculated in patients with cognitive data only. We explored the characteristics of the ORCHARD-EPR certain vs uncertain delirium groups using the standardised mean difference (SMD) to compare means of variables hypothesised to be related to delirium status based on the existing literature [1–3] (SMD > 0.1 considered clinically relevant).

Univariable odds ratios (ORs) and Wald p-values were calculated for binary outcomes for certain and uncertain delirium in ORCHARD-EPR and for delirium in the prospective cohorts. In multivariable analysis, confounding variables included age, sex, comorbidity (CCI; 0, 0–5, >5) [20] and illness severity (NEWS; Low, Low-Medium, Medium, High; SIRS≥2) [17, 18] and in a post-hoc analysis, frailty according to the modified HFRS [19]. Analyses were done in R version 4.2.1 [21].

### Declaration of sources of funding

This work was supported by the Rhodes Trust and CIHR Doctoral Foreign Study Award (ELB), Medical Research Council doctoral training fellowship (JMG), Nuffield Department of Population Health (SS), the NIHR Oxford NIHR Biomedical Research Centre and NIHR Invention for Innovation programme grant NIHR204290 (STP). The funders had no role in the design and conduct of the study, interpretation of the data or decision to submit the manuscript for publication.

## Results

Among 31,281 general medicine admissions aged ≥70 years in ORCHARD-EPR, 24 527 (78%) had a cognitive assessment (completed cognitive screen or ICD-10 code for delirium or dementia). Of these 18 614 (60%) were first admissions (mean/SD age = 82.9/7.4 years, female = 53%; Table 1) of whom 14 002 (75%) had a cognitive assessment (cognitive screen proforma or ICD-10 coding for delirium or dementia, Appendix 2 and 3). Patients with missing vs available cognitive data (*N* = 4612/18 614) were younger with less comorbidity and frailty, shorter admissions and lower mortality (all *P* < 0.001; Appendix Table 2). In the prospective cohorts, cognitive screening data were available for all 893 admissions aged ≥70 years of which 731 (82%) were first admissions (mean/SD age = 82.7/7.1 years). Demographics, clinical characteristics and in-hospital death in the two cohorts were similar (Table 1). The HFRS was greater in ORCHARD-EPR than in the prospective cohorts but similar when the latter were restricted to the years that overlapped with ORCHARD-EPR (2015-, *n* = 514, *P* = 0.3; Appendix Table 3).

**Figure 1:**
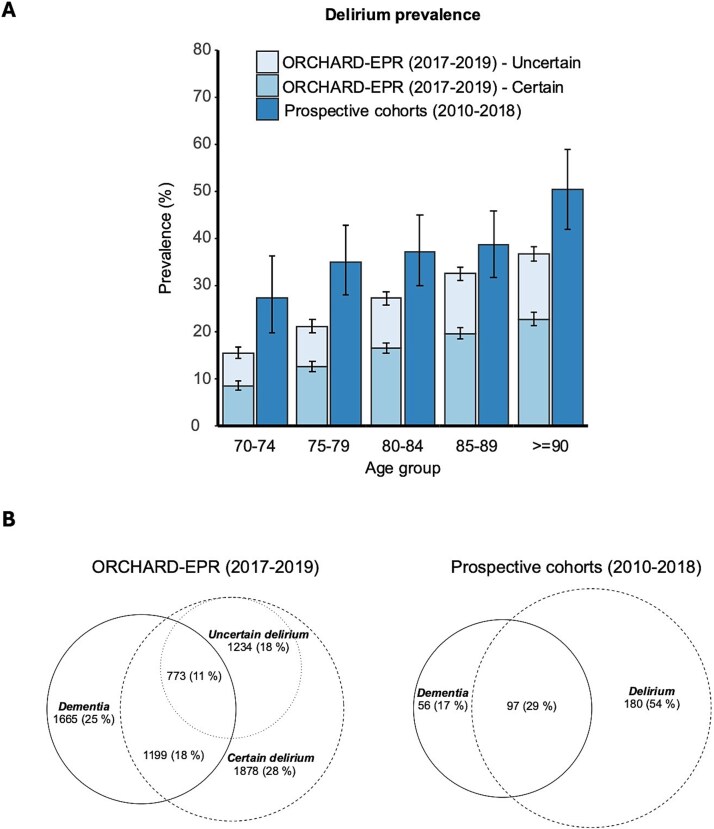
A) Prevalence of certain and uncertain delirium based on the CAM and holistic assessment supplemented with ICD-10 coding in ORCHARD-EPR 2017–2019 vs delirium by DSM-IV criteria in the prospective cohorts (2010–2018) by age. B) Proportions with delirium only, delirium superimposed on dementia and dementia only in the prospective cohorts and ORCHARD-EPR (of those with a delirium or dementia diagnosis).

The ascertainment of delirium and dementia in ORCHARD-EPR according to the EPR cognitive screen responses and the allocated ICD-10 codes is shown in Appendix Table 4. The prevalence of certain delirium was 3077/18 614 (17%, 95%CI 16%–17%) and of uncertain delirium was 2007/18 614 (11%,10%–11%) whereas the prevalence of delirium by DSM-IV criteria in the prospective cohorts was higher at 277/731 (38%, 34%–42%, both *P* < 0.001) (Figure 1A). In ORCHARD-EPR, pre-existing dementia prevalence was 3637/18,614 (20%,19%–20%) compared to 153/731 (21%,18%–24%) in the prospective cohorts (*P* = 0.4). Restricting to only patients with cognitive data in ORCHARD-EPR (*n* = 14,002), gave prevalences of certain delirium = 21%, uncertain delirium = 14% and dementia = 26%. The proportions with delirium, delirium superimposed on dementia, and dementia were similar in both cohorts (Figure 1B).

**Figure 2:**
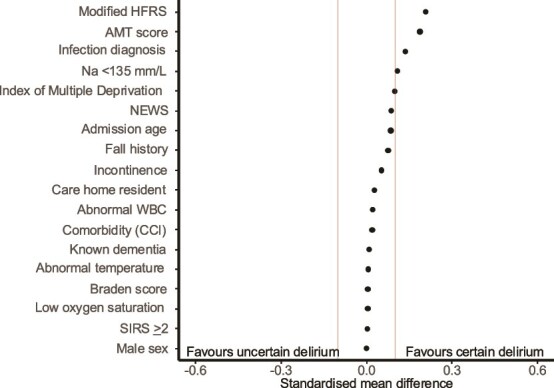
Standardised mean difference (SMD) for mean values of variables hypothesised to be related to uncertain vs certain delirium diagnosis in ORCHARD-EPR (2017–2019). Vertical red lines mark a standardised difference of $\pm$0.1. The HFRS was modified to exclude coded delirium (F05X, F1X4) to avoid introducing bias by including the independent variable in a dependent variable. CCI = Charlson Comorbidity Index. IMD = Index of Multiple Deprivation. Modified HFRS = Modified Hospital Frailty Risk Score. Incontinence = Incontinence or urinary urgency. Infection diagnosis = primary diagnosis of infection. SIRS = Systemic Inflammatory Response Syndrome criteria. Na = Serum Sodium. NEWS = National Early Warning Score.

In ORCHARD-EPR, the clinical characteristics of certain and uncertain delirium were broadly similar to each other and to delirium in the prospective cohorts (Appendix Tables 5 and 6). Certain and uncertain delirium groups were equally likely to have comorbid dementia (SMD = 0.009, Figure 2), but the group with certain delirium was slightly more likely to have a higher HFRS, low AMT score, infection and hyponatraemia (Na < 135 mm/L, SMD all>0.1).

**Figure 3:**
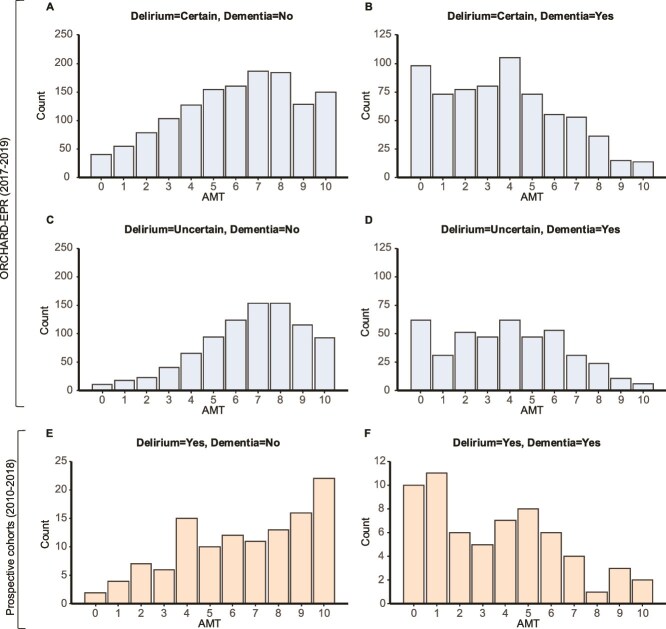
Histograms showing the distribution of AMT (range 0–10) scores in ORCHARD-EPR and prospective cohorts by delirium and dementia status. For ORCHARD-EPR, AMT scores for certain (A, B) and uncertain delirium (C,D) in the absence and presence of comorbid dementia are shown. For the prospective cohorts, AMT scores for delirium in the absence and presence of comorbid dementia are shown (E,F). Lower AMT scores indicate greater impairment.

The AMT was not feasible to perform in 2565/18,614 (14%) in ORCHARD-EPR and 88/731 (12%) in the prospective cohorts (p = 0.2) and the distribution of AMT scores was similar in both cohorts (median [IQR] AMT = 9 [7–10], vs median [IQR] AMT = 9 [6–10], Appendix Figure 3). AMT scores for those with certain and uncertain delirium in ORCHARD-EPR were similar (median [IQR] AMT = 5 [3–8], and median [IQR] AMT = 6 [4–8]) to the AMT scores for those with delirium (median [IQR] AMT = 5 [3–8]) in the prospective cohorts. Scores were lower for delirium superimposed on dementia than for delirium alone (Figure 3). Low AMT score in the absence of either a delirium or dementia diagnosis was present in 1078 (5.8%) in ORCHARD-EPR vs 39 (5.3%) in the prospective cohorts (*P* = 0.7). Prevalence of any cognitive frailty (delirium, dementia or low AMT) was lower in ORCHARD-EPR at 6534 (35%, 95%CI 34%–36%) than in the prospective cohorts (365, 50%, 46%–54%). However, the clinical characteristics (eg age, sex, illness severity, and care home residence) of the different cognitive frailty subgroups were broadly similar (Appendix Table 7).

Compared to those without delirium, patients with delirium in the prospective cohorts had an increased risk of in-hospital death (delirium 47/277,17%) vs without delirium 27/454 (6%, OR_adj_ = 2.29,1.62–4.56) which was similarly increased in ORCHARD-EPR for both certain and uncertain delirium (Table 2, Appendix Table 8). The excess mortality in uncertain and certain delirium was maintained at one year overall although neither was associated with death at one year in those with comorbid dementia.

In ORCHARD-EPR, compared with no delirium, length of stay>7 days, delayed discharge and discharge destination other than home was higher for certain and uncertain delirium irrespective of the presence of comorbid dementia (Table 2, Appendix Table 8). Readmission within 30 days of discharge was higher for those with certain delirium (361/2485 [15%]; OR_adj_ = 1.15, 1.02–1.31; *P* = 0.027) versus no delirium overall (1428/11,910 [12%]), but not in the subgroup with comorbid dementia (134/976 [14%], OR_adj_ 0.97, 0.78–1.22; *P* = 0.8). Uncertain delirium was not associated with increased risk of readmission irrespective of the presence or absence of comorbid dementia. Post-hoc analysis suggested that greater frailty explained the increased proportion with certain vs uncertain delirium who had length of stay>7 days, delayed discharge, discharge destination other than home and readmission (Appendix Table 9).

## Discussion

We examined real-world individual ORCHARD-EPR data following large scale implementation of cognitive assessment for older acute hospital patients. Delirium diagnosis, informed by the CAM in conjunction with the 10-point AMT and clinical assessment, was achieved in almost 80% of general medicine patients aged 70 years or older. Notably, clinicians were often uncertain in assigning a delirium diagnosis and the prevalence of certain delirium was therefore lower than in the prospective cohorts. Low cognitive score, infection, hyponatraemia and frailty were associated with increased clinician delirium diagnostic certainty whereas co-morbid dementia did not have an impact. Compared to no delirium, outcomes were poor in both certain and uncertain delirium and similar to the prospective cohorts although with larger effect sizes in certain delirium for length of stay, delayed discharge and discharge destination.

Real-world studies on the implementation of cognitive screening at scale in unplanned admissions are limited despite current guidelines and policy recommendations [1, 4–6, 22]. A large-scale study in general medicine patients ≥65 years with delirium diagnosis on admission using CAM and clinical assessment as in our study, reported a delirium prevalence of 25% but used additional specially trained nurses rather than the clerking resident doctors [23]. Delirium screening in routine practice using the 4AT (49%–77% screened) [24] demonstrated feasibility but prevalence in acute medicine was lower than expected (14%) when both screened and unscreened patients were included. Screening rates were higher at over 90% in a large registry study of hip fracture patients where 20% had scores suggestive of delirium [25]. Others have used machine learning or risk stratification to target screening in those at-risk [26, 27]. However, no previous study recorded delirium diagnosis as part of a holistic screening process, examined delirium diagnostic uncertainty, performed additional objective cognitive testing or compared with prospectively acquired reference data from the same setting.

**Table 2:** Outcomes of uncertain delirium and certain delirium groups vs those with no delirium stratified by the presence or absence of comorbid dementia in ORCHARD-EPR, adjusted for age, sex, comorbidity (Charlson comorbidity index category) and illness severity (NEWS category)

	Dementia							No dementia						
	No delirium (OR = 1)	Certain delirium (Delirium = yes or delirium ICD-10 code)			Uncertain delirium (Delirium = uncertain and no delirium ICD-10 code)			No delirium (OR = 1)	Certain delirium (Delirium = yes or delirium ICD-10 code)			Uncertain delirium (Delirium = uncertain and no delirium ICD-10 code)		
Outcome	Events/ N	Events/ N	Adjusted OR 95% CI	p-value	Events/ N	Adjusted OR 95% CI	p-value	Events/ N	Events/N	Adjusted OR 95% CI	p-value	Events/N	Adjusted OR 95% CI	p-value
In hospital death	160/ 1638	158/ 1183	1.44 1.13, 1.84	0.003	127/ 763	1.7 1.30, 2.20	<0.001	859/11 692	290/ 1858	2 1.72, 2.33	<0.001	184/ 1219	1.94 1.62, 2.32	<0.001
Death by 30 days	113/ 1478	102/ 1025	1.34 1.00, 1.77	0.046	80/ 636	1.7 1.25, 2.31	<0.001	542/10 833	103/ 1568	1.14 0.91, 1.42	0.2	83/ 1035	1.46 1.14, 1.86	0.002
Death 1 year	331/ 1365	242/ 923	1.11 0.92, 1.35	0.3	155/ 556	1.21 0.96, 1.51	0.1	1749/10 291	345/ 1465	1.33 1.16, 1.52	<0.001	212/ 952	1.27 1.07, 1.49	0.005
LoS >7 days	451/ 1638	587/ 1183	2.58 2.21, 3.03	<0.001	291/ 763	1.64 1.37, 1.97	<0.001	2778/11 692	873/ 1858	2.65 2.39, 2.94	<0.001	508/ 1219	2.17 1.92, 2.46	<0.001
Delayed discharge >2 days	206/ 1478	274/ 1025	2.25 1.84, 2.76	<0.001	110/ 636	1.29 1.00, 1.67	0.046	950/10,833	362/ 1568	2.76 2.40, 3.17	<0.001	201/ 1035	2.31 1.94, 2.73	<0.001
Discharge to usual place of residence	350/ 1457	386/ 1001	1.98 1.67, 2.37	<0.001	181/ 623	1.29 1.05, 1.60	0.017	1393/10 833	459/ 1515	2.54 2.24, 2.87	<0.001	261/ 1014	2.14 1.83, 2.49	<0.001
Readmission <30 days	202/ 1434	134/ 976	0.97 0.76, 1.22	0.8	89/ 624	1 0.76, 1.31	>0.9	1226/10 476	227/ 1509	1.27 1.09, 1.48	0.002	126/ 1006	1.03 0.84, 1.25	0.8

Abbreviations: OR = Odds Ratio. CI = Confidence Interval. LoS = Length of stay in hospital. Patients without cognitive data (no cognitive screen and no ICD-10 code for delirium or dementia) were included in the analysis as patients without delirium or dementia

The reasons for delirium diagnostic uncertainty are unclear. Patients with uncertain delirium had similar characteristics and outcomes to the certain delirium group, but lack of informant history at first assessment, or opportunity to observe the patient over time may have impacted clinician confidence. Even combining certain and uncertain delirium, prevalence did not reach that of the prospective cohorts suggesting some delirium was missed potentially because of screening by non-specialist staff vs expert DSM-IV-based diagnosis in the prospective cohorts or failure to recognise new incident delirium because of a focus on on-admission screening. In addition, it is possible that some subsyndromal/uncertain delirium was defined as delirium in the prospective cohorts since there is some subjectivity in interpretation of the DSM-IV criteria. Further, the sensitivity of the CAM may be suboptimal particularly when administered by non-specialist nurses in routine practice [28]. However, this was mitigated in our study by the administration of the cognitive screen by the clerking doctor, the proforma design incorporating cognitive testing and documentation of diagnosis, and delirium diagnosis made using all available information, and not just the CAM. In addition, we adopted a conservative approach in calculating ORCHARD-EPR delirium prevalence including both patients with and without cognitive data. Delirium prevalence calculated including only those with cognitive data was higher, and similar to the prospective cohorts.

Our finding that uncertainty in delirium diagnosis was not associated with co-morbid dementia is perhaps surprising in view of previous reports [29]. However, uncertainty may have been offset by clinician awareness of delirium risk factors hence the increased clinician diagnostic confidence in the presence of frailty, low AMT score, infection and hyponatraemia [1–3]. Alternatively, these factors might have been linked to greater delirium severity making diagnosis more obvious. Although outcomes were broadly similar for certain and uncertain delirium, greater frailty in the certain delirium group might have contributed to the stronger associations with length of stay, delayed discharge and discharge destination other than home [30].

Strengths of our study include the comparison of large-scale real world hospital EPR data covering a population of >800 000 obtained after cognitive screening implementation, with prospectively acquired cohorts from the same setting filling a key knowledge gap [1]. There are some limitations. We were not able to distinguish between delirium on admission vs incident delirium in ORCHARD-EPR, and delirium was under-ascertained even after including those with uncertain diagnosis. It is likely that delirium was missed more often in milder and incident cases given on admission screening but ascertainment was substantially better than in standard administrative datasets [7, 8]. In addition, although ORCHARD-EPR and prospective cohorts were obtained from the same setting in the same institution and had similar demographic and clinical characteristics, there were nevertheless differences in the time frames that might have impacted delirium prevalence. Further, our study covered a single region in the UK and findings may not be generalisable to other settings or service models. Finally, our study was conducted prior to the covid pandemic and subsequent policy updates on delirium.

Our findings indicate that implementation of cognitive and delirium screening at scale in busy acute hospital services for older patients is feasible and valid supporting current guidance [5]. Delirium screening is necessary since widely used frailty screening instruments do not reliably capture cognitive syndromes [30]. Electronic delirium screening enables real-time identification of patients across the hospital, automated triggering of interventions, improvements in ICD-10 coding without loss of specificity, and use of large-scale data to inform policy and practice. [1, 16]

In conclusion, EPR-embedded cognitive screening was achieved at scale but when common associates of delirium were absent, clinicians were often uncertain in diagnosing delirium. Prevalence of certain delirium was therefore lower than in prospective cohorts from the same setting. However, the characteristics and outcomes of uncertain and certain delirium were broadly similar suggesting that uncertain delirium nevertheless identified a group at-risk. Our findings suggest that ORCHARD-EPR is sufficiently accurate for epidemiologic studies of delirium and combines the large scale of administrative health data with the sensitivity of small prospective studies but without selection bias. Expansion of ORCHARD-EPR is planned to inform clinical services in neighbouring regions in the UK.

## Supplementary Material

Supplement_Age_Ageing_Oct_2025

## Research Data Transparency and Availability

At present, ethics approval restrictions limit the use of data to researchers within the University of Oxford or Oxford University Hospitals NHS Foundation Trust. Requests for data should be made to Professor Sarah Pendlebury and will be considered on a case by case basis.
