# Supplementary material for: Delirium prevalence, diagnostic uncertainty and outcomes in ORCHARD-EPR: validation against prospective reference cohorts
Source: Age Ageing. 2025 Oct 16;54(10):afaf284. doi: 10.1093/ageing/afaf284 (PMC12530185; doi:10.1093/ageing/afaf284)
Supplement: Supplement_Age_Ageing_Oct_2025 [file supplement_age_ageing_oct_2025.docx]

**Appendix**

**Delirium prevalence, diagnostic uncertainty and outcomes in ORCHARD-EPR: validation against prospective reference cohorts**

Boucher EL, BHSc^1^; Gan JM, MBBS^1^; Lovett NG, MD^2^; Smith SC, FRCP^2^; Shepperd S, DPhil^3^; Pendlebury ST, FRCP, DPhil^1,2,4^

^1^Wolfson Centre for Prevention of Stroke and Dementia, Wolfson Building, Nuffield Department of Clinical Neurosciences, University of Oxford, UK

^2^Departments of Acute General (Internal) Medicine and Geratology, Oxford University Hospitals NHS Foundation Trust, UK

^3^Nuffield Department of Population Health, University of Oxford, UK

^4^NIHR Biomedical Research Centre, Oxford University Hospitals NHS Foundation Trust, UK

Address correspondence to: Professor Sarah Pendlebury, Wolfson Centre for Prevention of Stroke and Dementia, Wolfson Building, John Radcliffe Hospital, Oxford OX3 9DU

Email: [sarah.pendlebury@ndcn.ox.ac.uk](mailto:sarah.pendlebury@ndcn.ox.ac.uk)

Telephone: +44 1865 231603

**Contents**

Appendix 1: Literature review methods and results

Appendix Table 1, Studies reporting delirium prevalence in general medicine and hospital-wide cohorts

Appendix 2. Cognitive screen design and implementation and staff training

Appendix 3. ORCHARD-EPR ascertainment of delirium according to EPR cognitive screen proforma responses and ICD-10 coding for delirium.

Appendix Table 2. Demographic and clinical characteristics of acute general medicine admissions in ORCHARD-EPR with versus without cognitive data

Appendix Table 3. Demographic and clinical characteristics of ORCHARD-EPR and prospective cohorts restricted to the most recent years (2015-2018)

Appendix Table 4. Cross-tabulation of EPR cognitive screen proforma results vs ICD-10 coded delirium and dementia

Appendix Table 5. Demographic and clinical characteristics of ORCHARD-EPR by delirium status

Appendix Table 6. Demographic and clinical characteristics of prospective cohorts by delirium status

Appendix Table 7. Characteristics of groups with cognitive impairment in ORCHARD-EPR vs the prospective cohorts.

Appendix Table 8. Outcomes of no, certain and uncertain delirium

Appendix Table 9. Outcomes of no, certain and uncertain delirium including an interaction term for frailty status (modified HFRS)

Appendix Figure 1. CONSORT diagram for ORCHARD-EPR cohort

Appendix Figure 2. Cognitive data included in ORCHARD-EPR

Appendix Figure 3. Distribution of AMT scores in ORCHARD-EPR and prospective cohorts.

**Appendix 1- Literature Review**

Current guidelines recommend screening for delirium in hospitalised older adults to inform care but recognition and documentation of delirium remains poor. Implementation of delirium screening using validated tools at scale as part of standard clinical care is therefore required, but there are few real-world data. We searched MEDLINE and EMBASE from inception to 28/02/2024 using MeSH and Emtree terms and text keywords related to [Delirium], [Dementia], [Cognitive Impairment], [Cognitive Defect/Decline], [Cognitive Spectrum Disorder] and [Hospitalisation] and reference lists of relevant reviews.

Studies were included if they reported delirium ascertained using validated screening tools or diagnosed clinically in hospital, included mostly unplanned admissions (>70% as stated by the study author or inferred from the study description); and reported data for hospital-wide or general medicine admissions. Studies were excluded if they used diagnostic coding or chart review alone to ascertain delirium, were conducted in outpatient, emergency department, short-stay, geriatric, rehabilitation, mixed or specialty-specific settings; conducted in non-representative populations (diagnosis, clinical trial, risk profile, excluded patients with dependency, etc.); n<100; did not report cohort age and/or the full text was not available in English. Title-abstract (n=2,148) and full-text screening was done by one researcher (XX). Data extraction included study and participant characteristics, recruitment method, delirium ascertainment method and prevalence, including by specialty where available.

Results. Fourteen studies met the inclusion criteria.^1-14^ In twelve studies, delirium ascertainment was done by a dedicated research or clinical team. In the remaining two studies, delirium was ascertained at scale as part of standard clinical care. In the first (n=10,014), specially trained nurses administered the confusion assessment method (CAM) on admission as part of a broader older person assessment.^14^ In the second (n=22,323 acute medicine), the 4AT was administered routinely by the admitting clinician (usually a junior doctor).^2^ In both studies, delirium prevalence was somewhat lower than might have been expected. Delirium was binarised as present/absent based on the CAM or 4AT screening tools, with no option for assessors to document diagnostic uncertainty. This may have contributed to lower than expected estimates (ie because clinicians were reluctant to class a patient as delirious after a single assessment when uncertainty might have been expected to be relatively high). In addition, neither study supplemented the estimated delirium prevalence from on-admission screening with administrative diagnostic (ICD-10) coding to capture delirium arising later during admission or compared findings to reference cohorts. Delirium was associated with worse outcomes including increased mortality but adjustment for confounding did not include all of age, sex, comorbidity and illness severity.

**Appendix Table 1. Studies reporting delirium prevalence in general medicine and hospital-wide cohorts**

| **Study (n=14)** | **Country** | **N** | **Sampling  (% enrolled)** | **Age in years** | **Female (%)** | **Exclusion criteria** | **Delirium ascertainment** | **Details of ascertainment** | **Prev (%)** | **Prev dementia group (%)** |
| --- | --- | --- | --- | --- | --- | --- | --- | --- | --- | --- |
| Hospital-wide |  |  |  |  |  |  |  |  |  |  |
| Welch (2019)^1^ | UK | 1,507 | Convenience (63%) | 80 | 54% | Age <65 years, ICU, palliative, logistics | DSM-V if 4AT ≥4/12 | Assessed day of study by research team (P) | 15% (20% possible delirium) | 32% |
| General medicine |  |  |  |  |  |  |  |  |  |  |
| Anand (2022)^2^ | Lothian, UK | 43,946* | Consecutive (100%) | 79 | 56% | Age <65 years, elective, not acute medicine | 4AT ≥4/12 | On admission by clinician (C) | 14% | NA |
| Collins (2010)^3^ | UK | 710 | Consecutive (88%) | 83 | 59% | Age <70 years, elective, logistics, refused, non-English speaking | CAM | Assessed by psychiatrist or registrar <72 h of admission (P) | 16% | 50% |
| Dani (2017)^4^ | UK | 710 | Consecutive (NA) | 83 | 59% | Age <70 years, LoS <48 hours, non-English speaking | Clinical assessment including CAM | Assessed on admission by psychiatrist (P) | 10% | NA |
| Eeles (2010)^5^ | UK | 278 | Unclear, presumed consecutive (71%) | 83 | 58% | Age <75 years, no patient/proxy consent | DSM-IV | Assessed on admission and every 48 h by geriatrician (P) | 37% | 57% |
| Erkinjuntti (1986)^6^ | Finland | 2000 | Consecutive (100%) | 79 | 57% | Age <55 years | Clinical criteria | Assessed by internists (O) | 15% | 41% |
| Fortini (2014)^7^ | Italy | 560 | Consecutive (94%) | 80 | 50% | Age <65 years, no patient/proxy consent | CAM | Assessed daily by doctor (P) | 11% | NA |
| Gonzalez (2007)^8^ | Chile | 542 | Consecutive (NA) | 78 | 62% | Age <65 years, no patient/proxy consent, unable to do assessments (incl. coma, severe aphasia) | CAM | Assessed every 48 h by research team (P) | 35% | NA |
| Gottlieb (1991)^9^ | USA | 235 | Consecutive (33%) | 77 | 61% | Age <70 years, admitted for short stay or terminal care, ICU, coronary care, stepdown care, dialysis or chemotherapy admissions, repeat admissions, no consent | DSM-III | Assessed within ~24 of admission by research team; diagnoses confirmed by psychiatrist (P) | 20% | NA |
| Lakhan (2011^)10^ | Australia | 413 | Random (72%) | 82 | 55% | Age <70 years, LoS <48 hours, coronary or intensive care unit, palliative, transferred | DSM-IV | Assessed daily by nurses; diagnosis confirmed by geriatrician if available (P) | 18% | NA |
| Muresan (2016)^11^ | Ireland | 200 | Consecutive (93%) | 81 | 50% | Age <70 years, palliative, severe aphasia, intubation, sensory problems, non-English speaking, no patient/proxy consent or consultant assent | CAM and DRS-R-98 | Assessed day of enrolment and days 3, 7 and 10 by research team (P) | 23% | NA |
| Pendlebury (2015)^12^ | UK | 503 (308 >65 years) | Consecutive (100%) | 72 (81 >65 years) | 52% (54% >65 years) | None | DSM-IV, informed by CAM | Assessed by clinician on admission, reviewed daily by consultant geriatrician (P) | 20%, all adults  (31% >65 years) | 51%  (NA for <65 years) |
| Praditsuwan (2013)^13^ | Thailand | 225 | Consecutive (50%) | 78 | 49% | Age <70 years, intubated, unable to communicate, uncooperative, transferred or too unwell | DSM-IV | Assessed on admission and every 48 h by geriatrician (P) | 49% | 72% |
| Reynish (2018)^)14^ | UK | 10,014 | Consecutive (79%) | 79 | 57% | Age <65 years, <1 year follow-up, no OPRAA assessment (incl. Length of stay <24 h, imminent death and ICU) | CAM or clinical diagnosis | Assessed on admission by specialist nurses (C) | 25% | 46% |

**Legend:** *total study n was 82,770, but the n for the relevant population of acute medicine patients from Lothian is included here.  CAM=Confusion Assessment Method. ICU=Intensive Care Unit. OPRAA=Older Person’s Routine Acute Assessment. Prev = Prevalence. NA = not available. See details of ascertainment: (P) = Prospective ascertainment. (C) = Routine clinical data. (O) = Other or unclear. Ascertainment tools: DSM = Diagnostic and Statistical Manual of Mental Disorders.

**References**

1. Welch C, McCluskey L, Chapman GE, et al. Delirium is prevalent in older hospital inpatients and associated with adverse outcomes: Results of a prospective multi-centre study on World Delirium Awareness Day. *BMC Med* 2019; **17**(1): 229.

2. Anand A, Cheng M, Ibitoye T, Maclullich AMJ, Vardy E. Positive scores on the 4AT delirium assessment tool at hospital admission are linked to mortality, length of stay and home time: two-centre study of 82,770 emergency admissions. *Age Ageing* 2022; **51**(3).

3. Collins N, Blanchard MR, Tookman A, Sampson EL. Detection of delirium in the acute hospital. *Age Ageing* 2010; **39**(1): 131-5.

4. Dani M, Owen LH, Jackson TA, Rockwood K, Sampson EL, Davis D. Delirium, Frailty, and Mortality: Interactions in a Prospective Study of Hospitalized Older People. *J Gerontol A Biol Sci Med Sci* 2018; **73**(3): 415-8.

5. Eeles EMP, Hubbard RE, White SV, O'Mahony MS, Savva GM, Bayer AJ. Hospital use, institutionalisation and mortality associated with delirium. *Age Ageing* 2010; **39**(4): 470-5.

6. Erkinjuntti T, Wikstrom J, Palo J, Autio L. Dementia among medical inpatients. Evaluation of 2000 consecutive admissions. *Arch Intern Med* 1986; **146**(10): 1923-6.

7. Fortini A, Morettini A, Tavernese G, Facchini S, Tofani L, Pazzi M. Delirium in elderly patients hospitalized in internal medicine wards. *Intern Emerg Med* 2014; **9**(4): 435-41.

8. Gonzalez M, Martinez G, Calderon J, et al. Impact of delirium on short-term mortality in elderly inpatients: a prospective cohort study. *Psychosomatics* 2009; **50**(3): 234-8.

9. Gottlieb GL, Johnson J, Wanich C, Sullivan E. Delirium in the Medically Ill Elderly: Operationalizing the DSM-III Criteria. *Int Psychogeriatr* 1991; **3**(2): 181-96.

10. Lakhan P, Jones M, Wilson A, Courtney M, Hirdes J, Gray LC. A Prospective Cohort Study of Geriatric Syndromes Among Older Medical Patients Admitted to Acute Care Hospitals. *J Am Geriatr Soc* 2011; **59**(11): 2001-8.

11. Muresan M-L, Adamis D, Murray O, O'Mahony E, McCarthy G. Delirium, how does it end? Mortality as an outcome in older medical inpatients. *Int J Geriatr Psychiatry* 2016; **31**(4): 349-54.

12. Pendlebury ST, Lovett NG, Smith SC, et al. Observational, longitudinal study of delirium in consecutive unselected acute medical admissions: age-specific rates and associated factors, mortality and re-admission. *BMJ Open* 2015; **5**(11): e007808.

13. Praditsuwan R, Sirisuwat A, Assanasen J, et al. Short‐term clinical outcomes in delirious older patients: A study at general medical wards in a university hospital in Thailand. *Geriatr Gerontol Int* 2013; **13**(4): 972-7.

14. Reynish EL, Hapca SM, De Souza N, Cvoro V, Donnan PT, Guthrie B. Epidemiology and outcomes of people with dementia, delirium, and unspecified cognitive impairment in the general hospital: prospective cohort study of 10,014 admissions. *BMC Med* 2017; **15**(1): 140.

**Appendix 2.**

**Cognitive screen design and implementation and staff training**

In designing our cognitive screen, we chose the Confusion Assessment Method (CAM, short version) as the most validated pragmatic delirium screen available at the time.^1^ The CAM requires the clinician to assess the patient for the presence of the following:

A. Acute onset/fluctuation for which collateral history is required.

B. Inattention: Does the patient have difficulty focusing attention?

C. Disordered thinking

D. Altered level of consciousness.

The CAM is positive if A and B are present and either C or D. However, the CAM is a screen rather than a diagnostic test and the clinician is therefore required to document delirium diagnosis on the basis of all available information and whether they are certain or uncertain about the diagnosis.

We combined the CAM with the 10-point abbreviated mental test (AMT) to identify and quantify the severity of cognitive deficits including in those without delirium having demonstrated that the AMT was more feasible to do than the mini-mental-state-examination (MMSE) in routine administration to all older patients admitted to an acute medicine service.^2^ We validated the AMT against the Montreal Cognitive Assessment (MoCA) in two studies which showed it was specific for moderate/severe impairment although insensitive to milder impairments.^3,4^

The cognitive screen was delivered initially via a paper clerking proforma (2012-2015) and from 2015, via a bespoke structured powerform (Cerner Millenium) integrated into the Electronic Patient Record (EPR).^5^  The EPR cognitive screen was triggered automatically on-admission for all patients aged >70 years with unplanned admission to ensure completion and also because most (80%) delirium in unplanned hospital admissions is present on admission rather than being incident during admission.^2^ Roll-out across all four general hospitals covering the Oxfordshire region (population ~800,000) was supported by a multicomponent intervention as described previously including staff training and performance feedback.^6^ Implementation and compliance with screening was further driven by the requirement to fulfil national dementia screening targets (2013-2020).^7^

The finalised screen included a cognitive test and two questions to document delirium or dementia diagnosis:

- The 10-point AMT, or reason for untestability recorded in a drop down multichoice list (eg too unwell, aphasia) including “other” where the problem was not listed with the option to record this using free text entry.
- “Does the patient have a known diagnosis of dementia?”,
- “Does the patient have delirium?” informed by the CAM (individual CAM items were not recorded).

The assessor was required to record their answer to the delirium question with one of the following: “yes”, “no” or “uncertain”. We enabled clinicians to record diagnostic uncertainty in the diagnosis of delirium to reflect clinical realities including difficulty in establishing recent behavioural change (a key component of the DSM criteria for delirium and therefore the CAM and the 4AT). The proforma was designed for completion even in untestable patients. For example, in a patient with severe delirium and agitation who was untestable with the AMT, the AMT would be recorded as not feasible because the patient was uncooperative, and the delirium diagnosis question would be answered as “yes”. Partial completion of the proforma was not possible.

Clinical best practice requires reassessment in patients with uncertain delirium diagnosis or if new behavioural change occurs during admission but the EPR cognitive screening form was not used routinely to document reassessment at the time of the study and free text entry via powernote was the default. However, around one fifth of those with uncertain delirium and 5% who initially screened negative subsequently received a delirium ICD-10 code suggesting that further clinical assessment for delirium with free text documentation of findings was fairly common.

Training in use of the cognitive screen including the CAM was provided by XXX supported by other senior clinicians with a relevant specialist background (general medicine, geratology, psychiatry) at regular resident teaching, medical student teaching and teaching of advanced nurse practitioners. The original CAM manual developed for the purposes of training those administering the CAM is lengthy and training in all aspects of the manual was not possible given resources constraints, and high staff turnover. The CAM was originally validated for use with observations made during patient interview supplemented by the mini-mental-state examination (MMSE). In our training in use of the CAM, we teach residents to use the clerking process (history and physical examination) to provide the interview information. The clerking process generally takes at least 20 minutes of direct engagement with the patient and therefore provides the ideal opportunity to observe the patient’s behaviour and responses in some detail. Since the short CAM version was used in the cognitive screen, training relevant to the longer CAM items was included as general context to the short version items (eg disorientation, perceptual disturbance, psychomotor agitation/retardation, altered sleep wake cycle). Importantly, training in the cognitive screen and in identification of delirium is done with reference to the DSM criteria for delirium and we make it explicit that the CAM is not a diagnostic test but rather is used to highlight the points to look for in assessing the patient. We also highlight that the CAM lacks sensitivity in particular for hypoactive delirium. Therefore the answer to the delirium question (yes/no/uncertain) must be made with reference to the DSM criteria and all available information and not just to the CAM.

We initially introduced the MMSE alongside the CAM in the first iteration of the cognitive screen (2010) but found this not to be feasible to administer routinely because of the length of time taken and subsequently this was changed to the 10-point Abbreviated Mental Test (AMT).^2^ The AMT includes orientation questions and a test of attention (counting backwards) which are particularly relevant to the CAM items. It should be noted that in the acute situation, there is a need to first identify a cognitive problem and the severity of deficits then to establish the underlying reason for the cognitive impairment. As well as delirium, patients may have dementia without delirium, in which a crude measure of severity according to the AMT score is clinically useful in informing care, or they may have undiagnosed cognitive impairment requiring investigation. Our cognitive screen was therefore developed accordingly rather than only for the purposes of identifying delirium.

Individual EPR data including the individual item datafields in the EPR cognitive screen were extracted by the hospital information analysts and entered by the research team into the Oxford Cognitive Comorbidity, Frailty and Ageing Research Database-Electronic Patient Records (ORCHARD-EPR) for analysis.^8^ Diagnostic (ICD-10) codes applied by the hospital administrative coding team after patient discharge were also extracted and entered into the database. ICD-10 diagnostic codes were included to supplement the cognitive screening results where free text recording of delirium had enabled the coding team to apply a code for delirium in the absence of a completed screen or where the screen had been completed but was initially negative for delirium or delirium was recorded as uncertain. Sensitivity for ICD-10 coding for delirium in our institution is good and considerably higher than in most other routinely acquired administrative datasets and specificity approaches 100%.^6^

It should be noted that identification of delirium in ORCHARD-EPR is therefore likely to be better for delirium present on admission (prevalent delirium) as compared to delirium occurring de novo during admission (incident) because of mandatory on admission screening. For incident delirium in which on admission screening was negative for delirium, this would only have been detected in ORCHARD-EPR where the clinical team had recognised and recorded the presence of delirium in free text entries in EPR since recording of incident delirium using the cognitive screening proforma was not done routinely. Delirium recorded as free text in EPR and subsequently coded with a delirium ICD-10 code post-discharge by the hospital coding team would therefore be detected in ORCHARD-EPR.

In contrast, in the prospective cohorts, the patients were seen at least every other day by the senior clinician (Consultant Physician equivalent to board certified specialist in the USA) and incident delirium was therefore prospectively ascertained in a systematic fashion with delirium diagnosis being made after discussion with the wider multidisciplinary team supplemented by the medical records and information from informants where appropriate.

**References**

1. Inouye SK, van Dyck CH, Alessi CA, Balkin S, Siegal AP, Horwitz RI. Clarifying confusion: the confusion assessment method. A new method for detection of delirium. Ann Intern Med.1990;113:941-8.

2. Pendlebury ST, Lovett NG, Smith SC, Dutta N, Bendon C, Lloyd-Lavery A, Mehta Z, Rothwell PM. Observational, longitudinal study of delirium in consecutive unselected acute medical admissions: age-specific rates and associated factors, mortality and re-admission. BMJ Open 2015;5(11):e007808.

3. Emery A, Wells J, Klaus SP, Mather M, Pessoa A, Pendlebury ST: Underestimation of Cognitive Impairment in Older Inpatients by the Abbreviated Mental Test Score versus the Montreal Cognitive Assessment: Cross-Sectional Observational Study. Dement Geriatr Cogn Disord Extra 2020;**10**:205-215.

4.Pendlebury ST, Klaus SP, Mather M, de Brito M, Wharton RM. Routine cognitive screening in older patients admitted to acute medicine: abbreviated mental test score (AMTS) and subjective memory complaint versus Montreal Cognitive Assessment and IQCODE. Age Ageing 2015;44:1000-5

5.

5. Boucher EL, Gan JM, Lovett NG, Smith SC, Shepperd S, Pendlebury ST. Implementation of Delirium Screening at Scale in Older Patients With Emergency Hospital Admission. JAMA Intern Med. 2025 May 27:e251128.

6. Pendlebury ST, Lovett NG, Thomson RJ, Smith SC. Impact of a system-wide multicomponent intervention on administrative diagnostic coding for delirium and other cognitive frailty syndromes: observational prospective study. Clin Med (Lond) 2020;20:454-464.

7. NHS England. Statistics: Dementia Assessment and Referral 2018-2019. No date. <https://www.england.nhs.uk/statistics/statistical-work-areas/dementia/dementia-assessment-and-referral-2018-19>.

8. Boucher E, Jell A, Singh S, et al. Protocol for the Development and Analysis of the Oxford and Reading Cognitive Comorbidity, Frailty and Ageing Research Database-Electronic Patient Records (ORCHARD-EPR). BMJ Open 2024;14:e085126.

**Appendix 3.**

**ORCHARD-EPR ascertainment of delirium according to EPR cognitive screen proforma responses and ICD-10 coding for delirium.**

For the 3077 certain delirium cases in ORCHARD-EPR, 783 (25%) had delirium=yes and ICD-10 code for delirium, 473 (15%) had delirium=uncertain and ICD-10 code, 431 (14%) had delirium=no and ICD-10 code and 390 (13%) without cognitive screen completion had ICD-10 code (see Appendix Table 4).

**Appendix Table 2. Demographic and clinical characteristics of acute general (internal) medicine admissions in ORCHARD-EPR (2017-2019) with versus without cognitive data (a completed cognitive screen and or >1 ICD-10 code(s) for dementia or delirium).**

|  | **First admissions ORCHARD-EPR 2017-2019 (N=18,614)** | | |
| --- | --- | --- | --- |
| **Characteristic** | **No, cognitive data**,  N = 4,612*^a^* (25%) | **Yes, cognitive data**,  N = 14,002^a^ (75%) | **p-value^b^** |
| Age | 80.8 (7.4) | 83.6 (7.2) | **<0.001** |
| Female sex | 2,381 / 4,612 (51%) | 7,393 / 14,002 (53%) | 0.054 |
| CCI | 7.1 (7.9) | 11.6 (10.9) | **<0.001** |
| ***Frailty markers*** |  |  |  |
| HFRS | 4.8 (4.3) | 8.9 (6.5) | **<0.001** |
| Fall history | 1,077 / 3,565 (30%) | 5,229 / 11,500 (45%) | **<0.001** |
| Incontinence | 411 / 3,557 (12%) | 3,060 / 11,540 (27%) | **<0.001** |
| Braden score | 19.0 (3.3) | 17.5 (3.6) | **<0.001** |
| ***Observations and labs*** |  |  |  |
| SIRS >2^c^ | 1,692 / 4,507 (38%) | 5,454 / 13,824 (39%) | 0.022 |
| Low oxygen saturation | 1,315 / 4,604 (29%) | 4,059 / 13,981 (29%) | 0.5 |
| CRP >6 mm/L | 2,912 / 3,968 (73%) | 10,165 / 13,026 (78%) | **<0.001** |
| Na <135 mm/L | 1,416 / 4,519 (31%) | 4,352 / 13,875 (31%) | 0.9 |
| Length of stay (days) | 2 (1, 5) | 4 (2, 11) | **<0.001** |
| In-hospital death | 319 / 4,612 (6.9%) | 1,496 / 14,002 (11%) | **<0.001** |
| Numbers are Mean (SD); n (%); Median (IQR) for length of stay. | | | |
|  | | | |
| ^a^Missing from denominator: No cognitive data = 614, Yes cognitive data =1,249  ^b^t-test; Pearson's Chi-squared test, Fisher's exact test, Wilcoxon rank sum test for length of stay. Significant p-values bolded (<0.01). | | | |
| ^c^P-values for each criterion: abnormal tympanic temperature (0.2), WCC (<0.001), HR>90 (0.4) and RR>20 (0.8) | | | |

**Appendix Table 3.** **Demographic and clinical characteristics of ORCHARD-EPR (2017-2019) and prospective cohorts restricted to the most recent years (2015-2018)**

| **Characteristic^a^** | **ORCHARD-EPR 2017-2019**,  N = 18,614 | **Prospective cohorts**  **2015-2018**,  N = 514 | **p-value^b^** |
| --- | --- | --- | --- |
| Age | 82.9/7.4 | 82.7 (7.1) | 0.5 |
| Female sex | 9,774 (53%) | 257 / 514 (50%) | 0.3 |
| CCI | 10.5 (10.4) | 11.1 (11.0) | 0.5 |
| ***Frailty markers*** |  |  |  |
| HFRS | 7.9 (6.3) | 7.7 (6.3) | 0.3 |
| Fall history | 6,306 /15,065 (42%) | 261 / 514 (51%) | <0.001 |
| Incontinence | 3,471 /15,097 (23%) | 170 / 514 (33%) | <0.001 |
| Braden score^c^ | 17.9/3.6 | 17.9 (3.6) | >0.9 |
| ***Observations and labs*** |  |  |  |
| SIRS >2 | 7,146 / 18,331 (39%) | 187 / 493 (38%) | 0.6 |
| Low oxygen saturation | 5,374 / 18,585 (29%) | 147 / 513 (29%) | 0.9 |
| CRP >6 mm/L | 13,077 / 16,994 (77%) | 350 / 482 (73%) | 0.026 |
| Na <135 mm/L | 5,768 / 18,394 (31%) | 160/511 (31%) | >0.9 |
| LoS (days) | 3 (1, 9) | 3 (1, 9) | 0.9 |
| In-hospital death | 1,815 / 18,614 (9.8%) | 59/514 (11%) | 0.2 |
| ^a^Numbers are mean/SD or n (%), except for length of stay for which Median (IQR) is shown. | | | |
| ^b^T-test; Pearson's Chi-squared test, Fisher's exact test or Wilcoxon rank sum test (for length of stay only). | | | |
| ^c^ORCHARD-EPR N=16,751, Prospective cohorts=468 | | | |

Appendix Table 4. Cross-tabulation of EPR cognitive screen proforma results vs ICD-10 coded delirium and dementia

| ***Cognitive screening proforma question*** | **Total admissions**  **N** | **Delirium ICD-10 code allocated by coding team after discharge**  **N (%)** |
| --- | --- | --- |
| **Does the patient have delirium?** |  | *Delirium: F05X, Other* |
| Yes | 1,783 | 951 (53) |
| Uncertain | 2,480 | 473 (19) |
| No | 8,400 | 431 (5) |
| Not done | 5,951 | 390 (7) |
| **Total** | **18,614** | **2,245 (12)** |
| ***Does the patient have a diagnosis of dementia?*** |  | *Dementia: F00X, F01X, F02X,  F03X, G03X, Other* |
| Yes | 2,180 | 1,992 (91) |
| Uncertain* | 1,068 | 204 (19) |
| No | 9,415 | 403 (4) |
| Not done | 5,951 | 850 (14) |
| **Total** | **18,614** | **3,449 (19)** |

*For the current paper, patients with dementia=uncertain without an ICD-10 dementia code were assigned as no dementia based on our previous work (Clinical Medicine 2020;20:454-464)..

**Appendix Table 5. Demographic and clinical characteristics of ORCHARD-EPR by delirium status**

| **Characteristic** | **ORCHARD-EPR,**  N = 18,614 | **Certain delirium,**  N = 3,077 | **Uncertain delirium,**  N = 2,007 | **No delirium,**  N = 13,530 | **p-value^a^** | **p-value^b^** |
| --- | --- | --- | --- | --- | --- | --- |
| Age | 82.9/7.4 | 85.1 (7.0) | 84.5 (7.1) | 82.1 (7.3) | <0.001 | 0.003 |
| Female sex | 9,774 (53%) | 1,693 / 3,077 (55%) | 1,104 / 2,007 (55%) | 6,977 / 13,530 (52%) | <0.001 | >0.9 |
| CCI | 10.5 (10.4) | 14.1 (11.6) | 13.8 (11.4) | 9.2 (9.6) | <0.001 | 0.5 |
| ***Frailty markers*** |  |  |  |  |  |  |
| Modified HFRS | 7.5 (5.9) | 11.2 (6.4) | 9.9 (6.1) | 6.3 (5.3) | <0.001 | <0.001 |
| Fall history | 6,306 /15,065 (42%) | 1,452 / 2,610 (56%) | 816 / 1,574 (52%) | 4,038 / 10,881 (37%) | <0.001 | 0.017 |
| Incontinence | 3,471 /15,097 (23%) | 1,063 / 2,640 (40%) | 601 / 1,596 (38%) | 1,807 / 10,861 (17%) | <0.001 | 0.092 |
| Braden score | 17.9/3.6 | 16.2 (3.4) | 16.2 (3.7) | 18.5 (3.4) | <0.001 | 0.9 |
| ***Observations and labs*** |  |  |  |  |  |  |
| SIRS >2 | 7,146 / 18,331 (39%) | 1,275 / 3,043 (42%) | 821 / 1,967 (42%) | 5,050 / 13,321 (38%) | <0.001 | >0.9 |
| NEWS >5 | 4,119 / 18,470 (22%) | 717 / 3,056 (23%) | 518 / 1,989 (26%) | 2,884 / 13,425 (21%) | <0.001 | 0.037 |
| CRP >6 mm/L | 13,077 / 16,994 (77%) | 2,461 / 2,964 (83%) | 1,528 / 1,889 (81%) | 9,088 / 12,141 (75%) | <0.001 | 0.057 |
| Na <135 mm/L | 5,768 / 18,394 (31%) | 1,004 / 3,054 (33%) | 553 / 1,983 (28%) | 4,211 / 13,357 (32%) | <0.001 | <0.001 |
| Infection diagnosis^c^ | 1,393 / 18,614(7.5%) | 371 / 3,077 (12%) | 160 / 2,007 (8.0%) | 862 / 13,530 (6.4%) | <0.001 | <0.001 |
| Numbers are mean/SD or n (%). | | | | | | |
| ^a^Pearson's Chi-squared test or Kruskal-Wallis test for no, uncertain and certain delirium. | | | | | | |
| ^b^Pearson's Chi-squared test or T-test for uncertain versus certain delirium.  ^c^Infection as primary diagnosis based on ICD-10 coding. | | | | | | |
| **Abbreviations:** CCI = Charlson Comorbidity Index. Modified HFRS = Hospital Frailty Risk Score excluding coded delirium. Incontinence = Incontinence or urinary urgency. Infection diagnosis = primary diagnosis of infection. SIRS = Systemic Inflammatory Response Syndrome criteria. CRP = C-Reactive Protein. Na = Serum Sodium. | | | | | | |

Appendix Table 6. Demographic and clinical characteristics of prospective (2010-2018) cohorts by delirium status

| **Characteristic^a^** | **Overall,**  N = 731 | **Delirium,** N = 277 | **No delirium,** N = 454 | **p-value^b^** |  |
| --- | --- | --- | --- | --- | --- |
| Age | 82.7/7.1 | 83.9 (7.1) | 82.0 (6.9) | <0.001 |  |
| Female sex | 389 (53%) | 154 / 277 (56%) | 235 / 454 (52%) | 0.3 |  |
| CCI | 10.7 (10.7) | 13.0 (11.9) | 9.3 (9.7) | <0.001 |  |
| ***Frailty markers*** |  |  |  |  |  |
| Modified HFRS | 6.5 (5.6) | 9.1 (6.0) | 4.9 (4.7) | <0.001 |  |
| Fall history | 333 / 730 (46%) | 161 / 277 (58%) | 172 / 453 (38%) | <0.001 |  |
| Incontinence | 231 / 729 (32%) | 142 / 276 (51%) | 89 / 453 (20%) | <0.001 |  |
| Braden score | 17.9/3.6 | 16.4 (3.7) | 18.9 (3.1) | <0.001 |  |
| ***Observations and labs*** |  |  |  |  |  |
| SIRS >2 | 189 / 496 (38%) | 83 / 198 (42%) | 106 / 298 (36%) | 0.2 |  |
| Low oxygen saturation | 147 / 513 (29%) | 58 / 206 (28%) | 89 / 307 (29%) | 0.8 |  |
| CRP >6 mm/L | 396 / 538 (74%) | 174 / 221 (79%) | 222 / 317 (70%) | 0.024 |  |
| Na <135 mm/L | 184 / 581 (32%) | 78 / 227 (34%) | 106 / 354 (30%) | 0.3 |  |
| ^a^Numbers are mean/SD or n (%). | | | | | |
| ^b^Two Sample t-test; Pearson's Chi-squared test for delirium and no delirium. | | | | | |
|  | | | | | |
|  | | | | | |

**Appendix Table 7.** Characteristics of groups with cognitive impairment in ORCHARD-EPR vs the prospective cohorts. Delirium is shown for combined certain and uncertain groups for ORCHARD-EPR.

| Variables | **Dementia only** | | | **Delirium only** | | | **Delirium superimposed on dementia** | | | **AMTS<8 in the absence of dementia or delirium** | | |
| --- | --- | --- | --- | --- | --- | --- | --- | --- | --- | --- | --- | --- |
|  | **ORCHARD-EPR 2017-2019, N = 2,691 (8.5%)** | **Prospective cohorts**  **2010-2018, N = 75 (8.4%)** | **p-value** | **ORCHARD-EPR**  **2017-2019, N = 5,515 (17%)** | **Prospective**  **cohorts**  **2010-2018, N = 215 (24%)** | **p-value** | **ORCHARD-EPR**  **2017-2019, N = 3,778 (12%)** | **Prospective**  **cohorts**  **2010-2018, N = 130 (15%)** | **p-value** | **ORCHARD-EPR**  **2017-2019, N = 1,078 (3.4%)** | **Prospective cohorts**  **2010-2018, N = 39 (4.4%)** | **p-value** |
| Age | 86 (81, 90) | 85 (80, 90) | 0.5 | 85 (79, 90) | 83 (78, 88) | 0.072 | 86 (82, 90) | 86 (79, 89) | 0.10 | 86 (80, 90) | 83 (76, 88) | 0.036 |
| Female | 1,484 (56%) | 47 (63%) | 0.2 | 2,937 (54%) | 127 (59%) | 0.12 | 2,074 (56%) | 68 (52%) | 0.5 | 604 (57%) | 24 (62%) | 0.6 |
| Number of ICD-10 codes | 11.0 (8.0, 14.0) | 9.0 (7.0, 13.0) | **0.003** | 12.0 (9.0, 16.0) | 11.0 (7.0, 14.0) | **<0.001** | 12.0 (9.0, 16.0) | 10.5 (7.0, 14.8) | **<0.001** | 11.0 (8.0, 15.0) | 8.0 (6.0, 11.5) | **<0.001** |
| CCI | 22 (18, 31) | 18 (18, 30) | **0.006** | 9 (0, 16) | 7 (0, 14) | 0.067 | 22 (18, 31) | 20 (18, 29) | 0.012 | 8 (0, 16) | 8 (0, 14) | 0.4 |
| HFRS | 9.4 (6.0, 14.1) | 9.2 (4.3, 13.1) | 0.12 | 10.3 (6.5, 14.8) | 8.4 (4.9, 13.8) | **<0.001** | 14 (9, 19) | 13 (9, 19) | 0.4 | 8.0 (4.6, 11.8) | 5.3 (1.9, 8.7) | **<0.001** |
| AMTS | 7.0 (5.0, 9.0) | 5.0 (3.0, 7.0) | **<0.001** | 7.00 (4.00, 8.00) | 7.00 (4.00, 9.00) | 0.087 | 4.00 (2.00, 6.00) | 3.00 (1.00, 5.00) | 0.4 | NA | NA | NA |
| Low SpO2 | 803 (30%) | 14 (25%) | 0.5 | 1,663 (30%) | 53 (32%) | 0.7 | 1,064 (28%) | 31 (29%) | 0.8 | 307 (28%) | 13 (43%) | 0.077 |
| Abnormal temperature | 684 (25%) | 11 (21%) | 0.4 | 1,587 (29%) | 44 (27%) | 0.5 | 1,015 (27%) | 38 (36%) | 0.042 | 304 (28%) | 8 (28%) | >0.9 |
| Abnormal WBC | 956 (36%) | 22 (30%) | 0.3 | 2,283 (42%) | 91 (43%) | 0.7 | 1,517 (41%) | 52 (40%) | 0.9 | 371 (35%) | 13 (33%) | 0.9 |
| CRP >6 mm/L | 1,945 (78%) | 33 (63%) | 0.010 | 4,453 (84%) | 152 (84%) | >0.9 | 3,000 (83%) | 80 (75%) | 0.040 | 823 (81%) | 22 (69%) | 0.087 |
| Na <135 mm/L | 699 (26%) | 12 (20%) | 0.3 | 1,925 (35%) | 72 (39%) | 0.3 | 885 (24%) | 24 (22%) | 0.8 | 341 (32%) | 8 (23%) | 0.3 |
| SIRS ≥ 2 | 993 (37%) | 19 (37%) | >0.9 | 2,344 (43%) | 62 (39%) | 0.3 | 1,531 (41%) | 47 (45%) | 0.5 | 414 (39%) | 12 (41%) | 0.8 |
| Braden score | 17.0 (14.0, 19.0) | 17.0 (14.0, 20.0) | 0.4 | 17.0 (14.0, 19.0) | 17.0 (14.0, 19.0) | 0.2 | 15.0 (13.0, 18.0) | 16.0 (13.0, 18.0) | 0.3 | 17.0 (15.0, 20.0) | 18.0 (15.0, 21.0) | 0.2 |
| Care home resident | 1,118 (42%) | 28 (37%) | 0.5 | 1,355 (25%) | 50 (23%) | 0.7 | 1,835 (49%) | 66 (51%) | 0.6 | 274 (25%) | 14 (36%) | 0.14 |
| Fall history | 1,139 (56%) | 46 (61%) | 0.3 | 2,326 (51%) | 116 (54%) | 0.5 | 1,761 (60%) | 96 (74%) | **0.002** | 478 (53%) | 22 (56%) | 0.7 |
| Incontinence | 719 (35%) | 39 (52%) | **0.002** | 1,620 (35%) | 90 (42%) | 0.049 | 1,513 (50%) | 94 (73%) | **<0.001** | 244 (27%) | 12 (31%) | 0.6 |
| Length of stay | 3 (1, 9) | 4 (2, 8) | 0.4 | 6 (3, 14) | 7 (3, 15) | 0.8 | 6 (2, 15) | 6 (2, 16) | >0.9 | 5 (2, 13) | 4 (2, 10) | 0.4 |
| In hospital death | 259 (9.6%) | 8 (11%) | 0.8 | 872 (16%) | 35 (16%) | 0.9 | 570 (15%) | 21 (16%) | 0.7 | 87 (8.1%) | 2 (5.1%) | 0.8 |
| ^a^Data shown as N (%) unless specified; mean (SD) for age, CCI, HFRS and Braden score; median (IQR) for length of stay | | | | | | | | | | | | |
| ^b^P-values obtained using Wilcoxon rank sum test; Pearson's Chi-squared test; or Fisher's exact test. | | | | | | | | | | | | |

**Appendix Table 8.** Outcomes of uncertain delirium and certain delirium groups vs those with no delirium in ORCHARD-EPR, adjusted for age, sex, comorbidity (Charlson Comorbidity Index category) and illness severity (NEWS category)

|  | **No delirium (OR=1)** | **Certain delirium**  (Delirium=yes or ICD-10 code) | | | **Uncertain delirium**  (Delirium=no or ICD-10 code) | | | **P-value certain vs uncertain^a^** |
| --- | --- | --- | --- | --- | --- | --- | --- | --- |
| Outcome | Events/N | Events/N | Adjusted OR  95% CI | p-value | Events/N | Adjusted OR  95% CI | p-value | p-value |
| ***Death*** |  | | |  |  |  |  |  |
| In hospital | 1,019 / 13,330 | 448 / 3,041 | 1.71  1.51, 1.94 | <0.001 | 311 / 1,982 | 1.79  1.55, 2.06 | <0.001 | 0.6 |
| 30 days | 655 / 12,311 | 205 / 2,593 | 1.24  1.05, 1.46 | 0.012 | 163 / 1,671 | 1.57  1.30, 1.88 | <0.001 | 0.034 |
| 1 year | 2,080 / 11,656 | 587 / 2,388 | 1.24  1.11, 1.38 | 0.001 | 367 / 1,508 | 1.24  1.09, 1.41 | <0.001 | >0.9 |
| ***Other*** |  |  |  |  |  |  |  |  |
| LoS >7 days | 3,229 / 13,330 | 1,460 / 3,041 | 2.62  2.41, 2.84 | <0.001 | 799 / 1,982 | 1.92  1.74, 2.12 | <0.001 | <0.001 |
| Delayed discharge >2 days | 1,156 / 12,311 | 636 / 2,593 | 2.76  2.47, 3.08 | <0.001 | 311 / 1,671 | 1.97  1.71, 2.26 | <0.001 | <0.001 |
| Discharge to usual place of residence | 1,743 / 12,138 | 845 / 2,516 | 2.60  2.36, 2.87 | <0.001 | 442 / 1,637 | 1.95  1.72, 2.20 | <0.001 | <0.001 |
| Readmission <30 days | 1,428 / 11,910 | 361 / 2,485 | 1.15  1.02, 1.31 | 0.027 | 215 / 1,630 | 1.03  0.88, 1.20 | 0.7 | 0.2 |

**Abbreviations:** OR = Odds Ratio. CI = Confidence Interval. LoS = Length of stay in hospital.

**Appendix Table 9.** Delirium outcomes (reference=no delirium) including an interaction term for a modified 108-item HFRS excluding delirium codes (F05X). Adjusted for age, sex, CCI, HFRS, NEWS. VIFs for all predictors were <5 for this model.

|  | **No delirium (OR=1)** | **Uncertain delirium** | | | | | **Certain delirium** | | | | | **P-value uncertain vs certain^2^** |
| --- | --- | --- | --- | --- | --- | --- | --- | --- | --- | --- | --- | --- |
| **Outcome** | **Events/N** | **Events/N** | **Crude OR^1^** | **Adjusted OR^1^** | **95% CI^1^** | **p-value** | **Events/N** | **Crude OR^1^** | **Adjusted OR^1^** | **95% CI^1^** | **p-value** | **p-value** |
| ***Death*** |  |  |  |  |  |  |  |  |  |  |  |  |
| In hospital | 1010/13267 | 312/1961 | 2.88 | 2.31 | 1.76, 3.04 | <0.001 | 446/3021 | 2.54 | 2.19 | 1.71, 2.79 | <0.001 | 0.7 |
| 30 days | 650/12257 | 159/1649 | 2.12 | 1.71 | 1.20, 2.41 | 0.003 | 205/2575 | 1.6 | 1.3 | 0.93, 1.80 | 0.12 | 0.2 |
| 1 year | 2074/11607 | 365/1490 | 1.41 | 1.18 | 0.92, 1.50 | 0.2 | 579/2370 | 1.72 | 1.46 | 1.18, 1.79 | <0.001 | 0.2 |
| ***Other*** |  |  |  |  |  |  |  |  |  |  |  |  |
| LOS >7 days | 3208/13267 | 786/1961 | 2.07 | 2.04 | 1.67, 2.49 | <0.001 | 1449/3021 | 2.63 | 2.62 | 2.20, 3.11 | <0.001 | 0.045 |
| DTOC >2 days | 1144/12257 | 306/1649 | 2.85 | 2.76 | 2.07, 3.66 | <0.001 | 631/2575 | 4.24 | 4.02 | 3.18, 5.05 | <0.001 | 0.024 |
| Discharge to new residence | 1738/12085 | 432/1617 | 2.49 | 2.35 | 1.84, 2.98 | <0.001 | 843/2498 | 3.04 | 2.71 | 2.21, 3.31 | <0.001 | 0.3 |
| Readmission <30 days | 1434/12257 | 214/1649 | 1.26 | 1.18 | 0.88, 1.56 | 0.3 | 360/2575 | 1.78 | 1.66 | 1.30, 2.10 | <0.001 | 0.055 |

**Appendix Figure 1. CONSORT diagram for ORCHARD-EPR cohort**

Adult OUHFT attendances included in ORCHARD-EPR 2017-2019 (n=101,787)

Length of stay ≥1 day (n=60,072)

Age ≥70 years (n=51,260)

Admissions, hospital-wide (n=51,202)

Inpatient length of stay <1 day (n=6,010)

Age <70 years (n=8,812)

No diagnostic codes recorded (n=58)

Admissions, including ambulatory emergency care (n=96,319)

Transferred or triaged without admission (n=5,438)

Inpatient admissions to OUHFT hospitals (n=66,082)

Ambulatory care (n=25,460); OR

Treated in community (n=4,777)

General (internal) medicine admissions (n=31,281)

Admitted to other specialties (n=19,921)

Unique patients/first admissions (n=18,614)

Repeat admissions (n=12,667)

**Note:** N=18,353 admissions for which complete data (age, sex, comorbidity and NEWS score) were available were included in main outcomes analyses.

**Appendix Figure 2. Cognitive data included in ORCHARD-EPR**

**
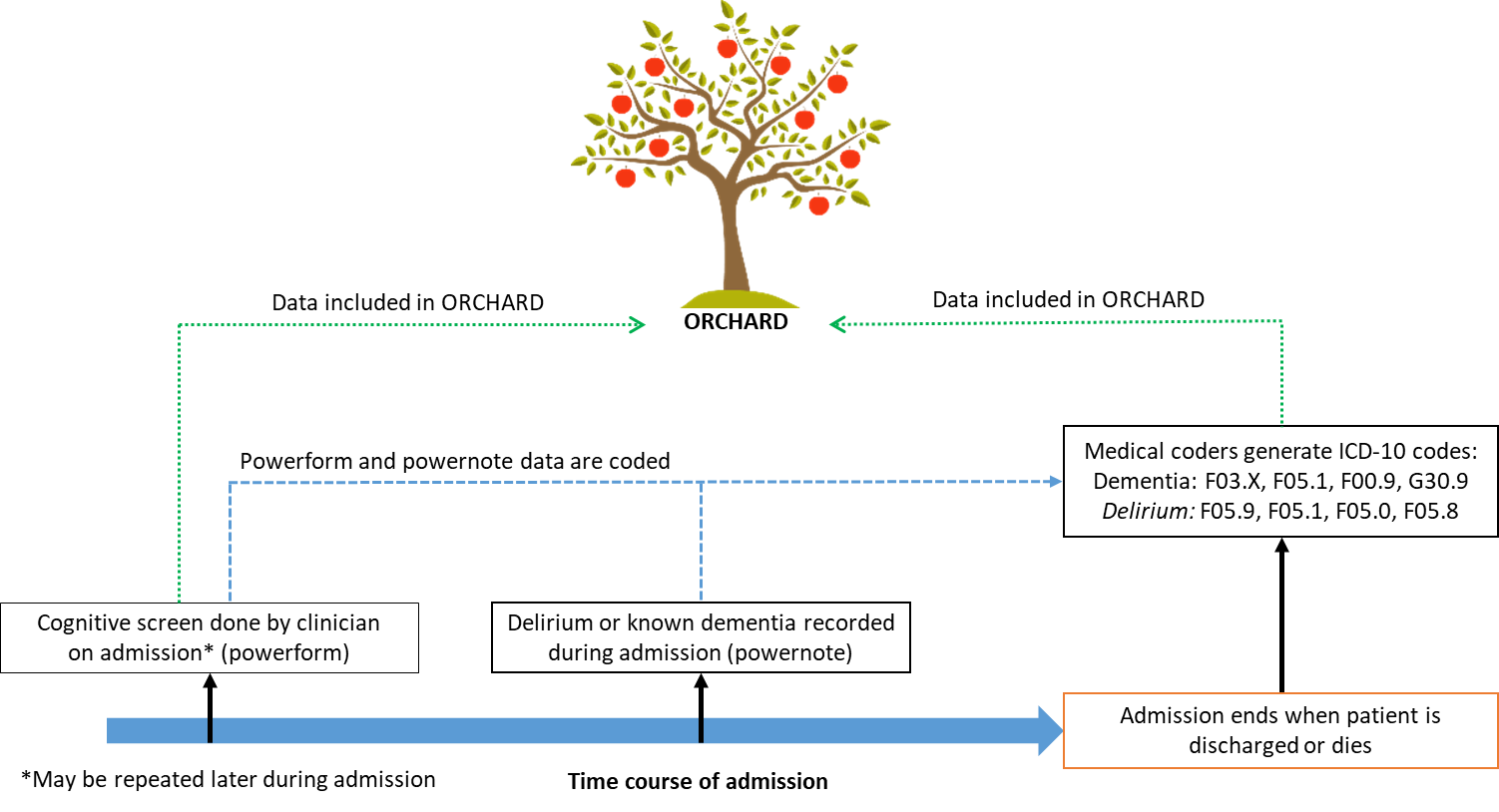
**

**Appendix Figure 3. Distribution of AMT scores in ORCHARD-EPR (2017-2019) and prospective cohorts (2010-2018)**

**
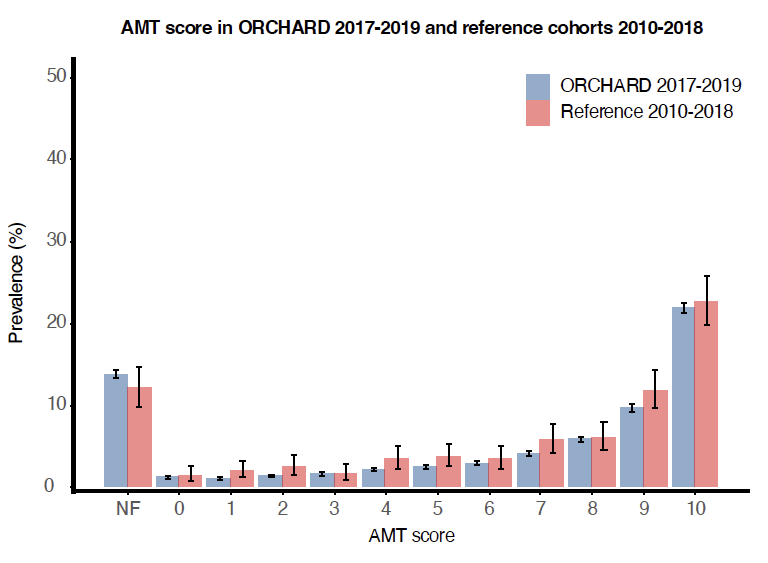
**

**Abbreviations:** NF = not feasible to do the AMT.

The reasons for untestability with the AMT are recorded in a drop down list in the EPR cognitive screening proforma (see Boucher et al. Implementation of Delirium Screening at Scale in Older Patients With Emergency Hospital Admission. JAMA Intern Med. 2025:e251128). Note that untestability with the AMT does not mean that the cognitive screening proforma is not completed – the cognitive screening proforma is completed irrespective of testability of otherwise on the AMT.
